# 2-(*N*-Cyclo­hexyl­carbamo­yl)benzene­sulfonamide

**DOI:** 10.1107/S1600536812000633

**Published:** 2012-01-14

**Authors:** Waseeq Ahmad Siddiqui, Adnan Ashraf, Hamid Latif Siddiqui, Muhammad Akram, Masood Parvez

**Affiliations:** a Department of Chemistry, University of Sargodha, Sargodha, Pakistan; b Institute of Chemistry, University of the Punjab, Lahore 54590, Pakistan; c Department of Chemistry, The University of Calgary, 2500 University Drive NW, Calgary, Alberta, Canada T2N 1N4

## Abstract

The asymmetric unit of the title compound, C_13_H_18_N_2_O_3_S, contains two mol­ecules with similar conformations. In both mol­ecules, the cyclo­hexyl rings adopt chair conformations, with the attached N atom in an equatorial orientation and an intra­molecular N—H⋯O hydrogen bond generates an *S*(7) ring. In the crystal, N—H⋯O hydrogen bonds link the mol­ecules and a C—H⋯O hydrogen bond is also observed. The crystal studied was a racemic twin.

## Related literature

For the biological activity of benzene­sulfonamide derivatives, see: Petrov *et al.* (2006 ▶); Eatedal *et al.* (2002 ▶); Ahmad *et al.* (2010 ▶). For related structures, see: Siddiqui *et al.* (2007 ▶, 2008 ▶). For ring puckering parameters, see: Cremer & Pople (1975 ▶).
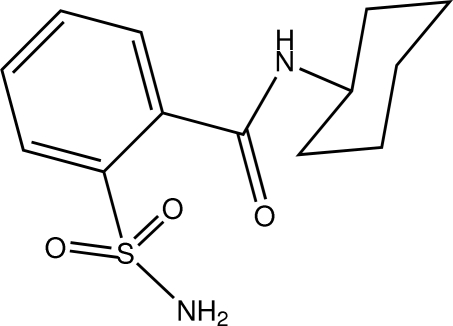


## Experimental

### 

#### Crystal data

C_13_H_18_N_2_O_3_S
*M* *_r_* = 282.35
Orthorhombic, 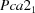

*a* = 16.1869 (5) Å
*b* = 10.8467 (3) Å
*c* = 15.9353 (4) Å
*V* = 2797.83 (13) Å^3^
*Z* = 8
Mo *K*α radiation
μ = 0.24 mm^−1^
*T* = 173 K
0.20 × 0.14 × 0.08 mm

#### Data collection

Nonius KappaCCD diffractometer
Absorption correction: multi-scan (*SORTAV*; Blessing, 1997 ▶) *T*_min_ = 0.954, *T*_max_ = 0.981
5929 measured reflections
5929 independent reflections
5451 reflections with *I* > 2σ(*I*)
*R*_int_ = 0.034

#### Refinement

*R*[*F* ^2^ > 2σ(*F* ^2^)] = 0.053
*wR*(*F* ^2^) = 0.110
*S* = 1.14
5929 reflections
362 parameters
1 restraint
H atoms treated by a mixture of independent and constrained refinement
Δρ_max_ = 0.31 e Å^−3^
Δρ_min_ = −0.28 e Å^−3^
Absolute structure: Flack (1983 ▶)
Flack parameter: 0.52 (8)

### 

Data collection: *COLLECT* (Hooft, 1998 ▶); cell refinement: *DENZO* (Otwinowski & Minor, 1997 ▶); data reduction: *SCALEPACK* (Otwinowski & Minor, 1997 ▶); program(s) used to solve structure: *SHELXS97* (Sheldrick, 2008 ▶); program(s) used to refine structure: *SHELXL97* (Sheldrick, 2008 ▶); molecular graphics: *ORTEP-3 for Windows* (Farrugia, 1997 ▶); software used to prepare material for publication: *SHELXL97*.

## Supplementary Material

Crystal structure: contains datablock(s) global, I. DOI: 10.1107/S1600536812000633/hb6585sup1.cif

Structure factors: contains datablock(s) I. DOI: 10.1107/S1600536812000633/hb6585Isup2.hkl

Supplementary material file. DOI: 10.1107/S1600536812000633/hb6585Isup3.cml

Additional supplementary materials:  crystallographic information; 3D view; checkCIF report

## Figures and Tables

**Table 1:** Hydrogen-bond geometry (Å, °)

*D*—H⋯*A*	*D*—H	H⋯*A*	*D*⋯*A*	*D*—H⋯*A*
N1—H11*N*⋯O4^i^	0.92 (4)	2.05 (4)	2.935 (4)	161 (4)
N2—H2*N*⋯O6^ii^	0.91 (4)	1.96 (4)	2.874 (4)	175 (4)
N3—H32*N*⋯O2^iii^	0.88 (4)	2.23 (4)	2.943 (4)	138 (4)
C3—H3⋯O1^iv^	0.95	2.54	3.254 (4)	132
N1—H12*N*⋯O3	0.86 (4)	2.18 (4)	2.938 (4)	146 (4)
N3—H31*N*⋯O6	0.85 (4)	2.09 (4)	2.831 (4)	145 (4)
N4—H4*N*⋯O3	0.85 (4)	2.11 (4)	2.952 (4)	171 (3)

Symmetry codes: (i) 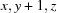; (ii) 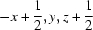; (iii) 
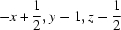
; (iv) 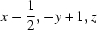.

**Fig. 1.**
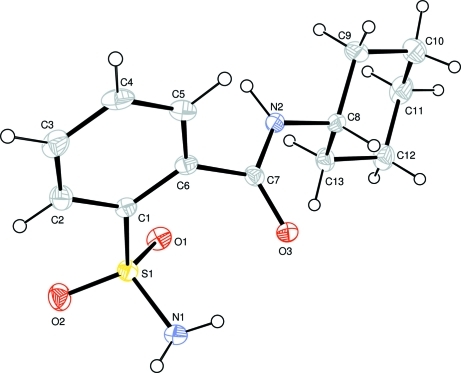
The molecule A of the title compound with displacement ellipsoids plotted at 30% probability level.

**Fig. 2.**
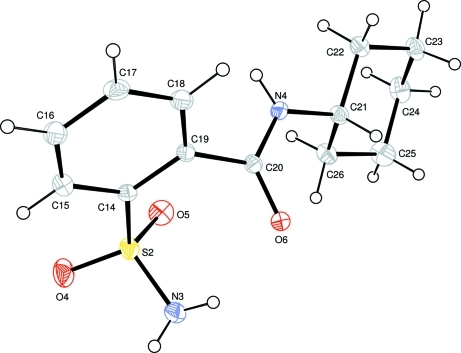
The molecule B of the title compound with displacement ellipsoids plotted at 30% probability level.

**Fig. 3.**
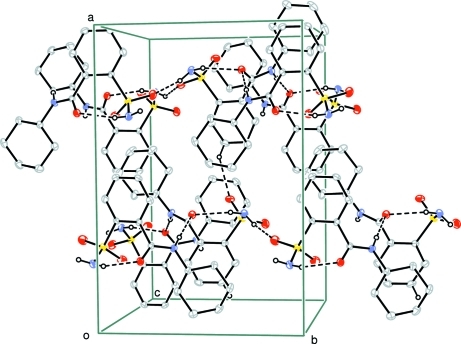
The unit cell packing diagram of the title compound showing hydrogen bonding interactions drawn with dashed lines. Hydrogen atoms not involved in H-bonds have been excluded for clarity.

## supplementary crystallographic information

### Comment

Derivatives of benzenesulfonamide find wide spread applications for the synthesis of pharmaceutical products which have bactericidal properties, various bioactive agents, artificial fibers, dyes, plasticizers and high molecular weight substances (Petrov *et al.*, 2006). Several pyrazole and oxadiazole derivatives have been reported to exhibit analgesic and anti-inflammatory activities and many drugs containing them are still in use in the market (Eatedal *et al.*, 2002). In continuation of our research on the synthesis of biological active benzothiazine derivatives (Siddiqui *et al.*, 2007) we report the synthesis and crystal structure of the title compound in this article.

There are two independent molecules in an asymmetric unit of the title compound, labeled as molecules A (Fig. 1) and B (Fig. 2) containing the S1 and S2 atoms, respectively. There are insignificant differences in the conformations of the two molecules, *e.g.*, the torsion angles C6–C7–N2–C8 and C19–C20–N4–C21 in molecules A and B are 177.3 (3) and -179.2 (3)°, respectively. In both molecules, the cyclohexyl rings adopt chair conformations with puckering parameters (Cremer & Pople, 1975) in molecules A and B being *Q* = 0.564 (4) and 0.573 (4) Å, θ = 3.1 (4) and 2.3 (4) ° and ω = 273 (8) and 251 (8) °, respectively. The bond distances and angles in both molecules agree very well with the cortresponding bond distances and angles reported in a closely related compound (Siddiqui *et al.*, 2007).

In the solid state, the molecules A and B are linked with each other *via* hydrogen bonds involving amino and O-atoms of the sulfonamide groups with N1···O4 = 2.935 (4) and N3···O2 = 2.943 (4) Å. The molecules A are linked into chains involving intermolecular interactions C3···O1 = 3.254 (4) Å. The molecules B do not show any such interactions. The molecules are stabilized by intramolecular interactions of the types N—H···O and C—H···O (Table 1 and Figure 3).

### Experimental

For the synthesis of the title compound, cyclohexylamine and saccharin were used as the starting materials following a reported procedure (Siddiqui *et al.*, 2008). Crystals of the title compound suitable for X-ray crystallographic study were grown from methanol at room temperature; m.p. = 512 – 513 K.

### Refinement

Though all the H atoms could be distinguished in the difference Fourier map the H-atoms bonded to C-atoms were included at geometrically idealized positions and refined in riding-model approximation with the following constraints: C—H = 0.95, 0.99 and 1.00 Å, for aryl, methylene and methine H-atoms, respectively. The *U*_iso_(H) were allowed at 1.2*U*_eq_(C). The hydrogen atoms bonded to the N-atoms were allowed to refine with *U*_iso_(H) = 1.2*U*_eq_(N). The final difference map was essentially featurless. The crystal was suggested by the program *SHELXL* (Sheldrick, 2008) to be a racemnic twin with a BASF twin factor 0.523 (8). The Friedel pairs (2644) of reflections were not merged.

### Crystal data

**Table:**

C_13_H_18_N_2_O_3_S	*F*(000) = 1200
*M**_r_* = 282.35	*D*_x_ = 1.341 Mg m^−^^3^
Orthorhombic, *P**c**a*2_1_	Mo *K*α radiation, λ = 0.71073 Å
Hall symbol: P 2c -2ac	Cell parameters from 3423 reflections
*a* = 16.1869 (5) Å	θ = 1.0–27.5°
*b* = 10.8467 (3) Å	µ = 0.24 mm^−^^1^
*c* = 15.9353 (4) Å	*T* = 173 K
*V* = 2797.83 (13) Å^3^	Prism, pale-yellow
*Z* = 8	0.20 × 0.14 × 0.08 mm

### Data collection

**Table:**

Nonius KappaCCD diffractometer	5929 independent reflections
Radiation source: fine-focus sealed tube	5451 reflections with *I* > 2σ(*I*)
graphite	*R*_int_ = 0.034
ω and φ scans	θ_max_ = 27.4°, θ_min_ = 2.6°
Absorption correction: multi-scan (*SORTAV*; Blessing, 1997)	*h* = −20→20
*T*_min_ = 0.954, *T*_max_ = 0.981	*k* = −14→14
5929 measured reflections	*l* = −20→20

### Refinement

**Table:**

Refinement on *F*^2^	Secondary atom site location: difference Fourier map
Least-squares matrix: full	Hydrogen site location: inferred from neighbouring sites
*R*[*F*^2^ > 2σ(*F*^2^)] = 0.053	H atoms treated by a mixture of independent and constrained refinement
*wR*(*F*^2^) = 0.110	*w* = 1/[σ^2^(*F*_o_^2^) + (0.009*P*)^2^ + 3.8318*P*] where *P* = (*F*_o_^2^ + 2*F*_c_^2^)/3
*S* = 1.14	(Δ/σ)_max_ < 0.001
5929 reflections	Δρ_max_ = 0.31 e Å^−^^3^
362 parameters	Δρ_min_ = −0.28 e Å^−^^3^
1 restraint	Absolute structure: Flack (1983)
Primary atom site location: structure-invariant direct methods	Flack parameter: 0.52 (8)

### Special details

**Table:**

Experimental. IR (KBr, max, cm^-1^) NH_2_ & NH 3318, 3275; CO 1680; SO_2_ 1320 and 1155; ^1^H-NMR (300 MHz, Methanol -d4) δ: 1.30–1.75 (m, 10H, cyclohexyl), 3.35 (m, 1H, cyclohexyl-CH), 5.55 (s, 2H, NH_2_), 7.73–8.13 (m, 4H, C_6_H_4_); ^13^C-NMR δ: 167.5, 137.5, 133.4, 131.9, 131.5, 127.7, 127.3, 45.7, 34.5, 27.3, 22.7
Geometry. All e.s.d.'s (except the e.s.d. in the dihedral angle between two l.s. planes) are estimated using the full covariance matrix. The cell e.s.d.'s are taken into account individually in the estimation of e.s.d.'s in distances, angles and torsion angles; correlations between e.s.d.'s in cell parameters are only used when they are defined by crystal symmetry. An approximate (isotropic) treatment of cell e.s.d.'s is used for estimating e.s.d.'s involving l.s. planes.
Refinement. Refinement of *F*^2^ against ALL reflections. The weighted *R*-factor *wR* and goodness of fit *S* are based on *F*^2^, conventional *R*-factors *R* are based on *F*, with *F* set to zero for negative *F*^2^. The threshold expression of *F*^2^ > σ(*F*^2^) is used only for calculating *R*-factors(gt) *etc*. and is not relevant to the choice of reflections for refinement. *R*-factors based on *F*^2^ are statistically about twice as large as those based on *F*, and *R*- factors based on ALL data will be even larger.

### Fractional atomic coordinates and isotropic or equivalent isotropic displacement parameters (Å^2^)

**Table:**

	*x*	*y*	*z*	*U*_iso_*/*U*_eq_	
S1	0.34082 (5)	0.60650 (7)	0.55872 (6)	0.02820 (16)	
S2	0.25588 (6)	−0.07073 (7)	0.31713 (6)	0.03103 (18)	
O1	0.40724 (14)	0.5355 (2)	0.59198 (15)	0.0357 (6)	
O2	0.31913 (17)	0.7220 (2)	0.59666 (17)	0.0440 (7)	
O3	0.36076 (14)	0.3676 (2)	0.43341 (13)	0.0287 (5)	
O4	0.27815 (17)	−0.1884 (2)	0.35130 (19)	0.0493 (7)	
O5	0.20767 (16)	0.0130 (2)	0.36603 (16)	0.0394 (6)	
O6	0.21853 (14)	0.1570 (2)	0.19541 (13)	0.0276 (5)	
N1	0.3621 (2)	0.6352 (3)	0.46194 (19)	0.0330 (7)	
H11N	0.327 (3)	0.690 (4)	0.437 (2)	0.040*	
H12N	0.369 (3)	0.568 (4)	0.434 (3)	0.040*	
N2	0.35953 (18)	0.2310 (2)	0.54146 (17)	0.0294 (6)	
H2N	0.335 (2)	0.212 (4)	0.591 (3)	0.035*	
N3	0.2057 (2)	−0.0976 (3)	0.2323 (2)	0.0355 (7)	
H31N	0.201 (3)	−0.034 (4)	0.202 (3)	0.043*	
H32N	0.222 (3)	−0.163 (4)	0.205 (3)	0.043*	
N4	0.25528 (18)	0.3051 (2)	0.28838 (17)	0.0260 (6)	
H4N	0.290 (2)	0.325 (3)	0.326 (2)	0.031*	
C1	0.25169 (19)	0.5111 (3)	0.5624 (2)	0.0257 (6)	
C2	0.1783 (2)	0.5611 (3)	0.5923 (2)	0.0353 (8)	
H2	0.1768	0.6444	0.6106	0.042*	
C3	0.1075 (2)	0.4899 (4)	0.5954 (3)	0.0407 (9)	
H3	0.0571	0.5248	0.6146	0.049*	
C4	0.1101 (2)	0.3689 (3)	0.5706 (3)	0.0392 (8)	
H4	0.0613	0.3203	0.5722	0.047*	
C5	0.1834 (2)	0.3174 (3)	0.5434 (2)	0.0339 (8)	
H5	0.1846	0.2328	0.5280	0.041*	
C6	0.2557 (2)	0.3867 (3)	0.53812 (19)	0.0269 (7)	
C7	0.3311 (2)	0.3281 (3)	0.5007 (2)	0.0262 (7)	
C8	0.4304 (2)	0.1578 (3)	0.5131 (2)	0.0295 (7)	
H8	0.4268	0.1483	0.4508	0.035*	
C9	0.4254 (2)	0.0303 (3)	0.5529 (3)	0.0418 (9)	
H9A	0.3743	−0.0114	0.5340	0.050*	
H9B	0.4227	0.0388	0.6147	0.050*	
C10	0.5003 (3)	−0.0486 (4)	0.5294 (3)	0.0558 (12)	
H10A	0.4980	−0.1273	0.5608	0.067*	
H10B	0.4979	−0.0681	0.4688	0.067*	
C11	0.5804 (3)	0.0149 (4)	0.5482 (3)	0.0551 (11)	
H11A	0.6267	−0.0367	0.5279	0.066*	
H11B	0.5864	0.0244	0.6097	0.066*	
C12	0.5848 (3)	0.1414 (4)	0.5067 (3)	0.0455 (9)	
H12A	0.5843	0.1317	0.4449	0.055*	
H12B	0.6369	0.1828	0.5227	0.055*	
C13	0.5111 (2)	0.2206 (3)	0.5341 (2)	0.0361 (8)	
H13A	0.5140	0.2353	0.5953	0.043*	
H13B	0.5136	0.3015	0.5054	0.043*	
C14	0.34848 (19)	0.0091 (3)	0.2919 (2)	0.0239 (6)	
C15	0.4234 (2)	−0.0517 (3)	0.3024 (2)	0.0346 (8)	
H15	0.4241	−0.1355	0.3196	0.042*	
C16	0.4974 (2)	0.0098 (3)	0.2877 (2)	0.0351 (8)	
H16	0.5486	−0.0319	0.2942	0.042*	
C17	0.4956 (2)	0.1325 (3)	0.2636 (2)	0.0327 (8)	
H17	0.5459	0.1750	0.2532	0.039*	
C18	0.4208 (2)	0.1936 (3)	0.2545 (2)	0.0296 (7)	
H18	0.4206	0.2782	0.2392	0.035*	
C19	0.34610 (19)	0.1327 (3)	0.26748 (18)	0.0227 (6)	
C20	0.26705 (18)	0.1994 (3)	0.24848 (18)	0.0221 (6)	
C21	0.1806 (2)	0.3805 (3)	0.2773 (2)	0.0264 (7)	
H21	0.1713	0.3936	0.2159	0.032*	
C22	0.1946 (2)	0.5054 (3)	0.3188 (2)	0.0324 (7)	
H22A	0.2100	0.4929	0.3783	0.039*	
H22B	0.2410	0.5479	0.2905	0.039*	
C23	0.1177 (2)	0.5864 (3)	0.3142 (2)	0.0354 (8)	
H23A	0.1275	0.6635	0.3460	0.042*	
H23B	0.1069	0.6088	0.2550	0.042*	
C24	0.0428 (2)	0.5213 (4)	0.3498 (3)	0.0426 (9)	
H24A	−0.0066	0.5740	0.3426	0.051*	
H24B	0.0509	0.5070	0.4106	0.051*	
C25	0.0290 (2)	0.3983 (4)	0.3057 (3)	0.0459 (10)	
H25A	−0.0192	0.3559	0.3309	0.055*	
H25B	0.0170	0.4128	0.2456	0.055*	
C26	0.1052 (2)	0.3169 (3)	0.3139 (3)	0.0346 (7)	
H26A	0.1151	0.2982	0.3739	0.042*	
H26B	0.0956	0.2381	0.2841	0.042*	

### Atomic displacement parameters (Å^2^)

**Table:**

	*U*^11^	*U*^22^	*U*^33^	*U*^12^	*U*^13^	*U*^23^
S1	0.0271 (4)	0.0283 (3)	0.0292 (4)	−0.0026 (3)	−0.0003 (3)	−0.0022 (3)
S2	0.0288 (4)	0.0271 (4)	0.0372 (4)	−0.0029 (4)	−0.0010 (4)	0.0083 (4)
O1	0.0255 (12)	0.0457 (14)	0.0359 (13)	−0.0016 (10)	−0.0031 (10)	0.0036 (11)
O2	0.0453 (15)	0.0363 (14)	0.0504 (16)	−0.0056 (12)	0.0067 (13)	−0.0126 (12)
O3	0.0335 (12)	0.0273 (11)	0.0253 (12)	0.0013 (9)	0.0015 (9)	0.0036 (9)
O4	0.0457 (16)	0.0363 (14)	0.0660 (19)	−0.0054 (12)	−0.0088 (14)	0.0238 (13)
O5	0.0355 (13)	0.0477 (15)	0.0351 (14)	−0.0036 (12)	0.0101 (11)	0.0036 (11)
O6	0.0307 (12)	0.0239 (11)	0.0282 (12)	0.0004 (9)	−0.0059 (9)	−0.0009 (9)
N1	0.0382 (17)	0.0278 (15)	0.0330 (16)	−0.0030 (13)	0.0030 (13)	0.0038 (12)
N2	0.0365 (16)	0.0249 (13)	0.0270 (15)	0.0062 (12)	0.0058 (12)	0.0034 (11)
N3	0.0340 (17)	0.0258 (15)	0.0467 (19)	−0.0033 (13)	−0.0067 (14)	0.0005 (13)
N4	0.0262 (13)	0.0235 (13)	0.0283 (14)	0.0039 (11)	−0.0071 (11)	−0.0022 (10)
C1	0.0236 (13)	0.0287 (14)	0.0247 (14)	−0.0002 (12)	0.0000 (13)	0.0030 (15)
C2	0.0307 (17)	0.0348 (18)	0.040 (2)	0.0048 (15)	0.0029 (15)	0.0011 (15)
C3	0.0252 (17)	0.052 (2)	0.045 (2)	0.0103 (16)	0.0064 (15)	0.0092 (17)
C4	0.0281 (17)	0.045 (2)	0.045 (2)	−0.0094 (15)	0.0008 (16)	0.0169 (18)
C5	0.0335 (18)	0.0302 (16)	0.038 (2)	−0.0062 (14)	−0.0009 (14)	0.0058 (14)
C6	0.0265 (15)	0.0297 (15)	0.0244 (16)	0.0004 (13)	−0.0024 (12)	0.0059 (12)
C7	0.0296 (17)	0.0225 (15)	0.0265 (16)	−0.0018 (13)	−0.0023 (13)	−0.0020 (12)
C8	0.0380 (19)	0.0266 (16)	0.0240 (16)	0.0057 (14)	0.0051 (13)	0.0027 (13)
C9	0.051 (2)	0.0281 (17)	0.046 (2)	0.0075 (15)	0.0052 (19)	0.0097 (17)
C10	0.075 (3)	0.035 (2)	0.057 (3)	0.024 (2)	0.013 (2)	0.0082 (19)
C11	0.054 (3)	0.060 (3)	0.051 (3)	0.033 (2)	0.009 (2)	0.007 (2)
C12	0.039 (2)	0.049 (2)	0.048 (2)	0.0136 (18)	0.0046 (18)	−0.0036 (19)
C13	0.0355 (19)	0.0340 (18)	0.0390 (19)	0.0084 (15)	0.0028 (15)	−0.0044 (15)
C14	0.0219 (14)	0.0214 (14)	0.0285 (16)	−0.0033 (12)	−0.0027 (12)	−0.0006 (11)
C15	0.0361 (19)	0.0261 (16)	0.042 (2)	0.0023 (14)	−0.0063 (15)	0.0001 (14)
C16	0.0244 (15)	0.0324 (17)	0.049 (2)	0.0046 (13)	−0.0031 (15)	−0.0066 (16)
C17	0.0232 (16)	0.0336 (17)	0.041 (2)	−0.0069 (14)	−0.0008 (14)	−0.0046 (15)
C18	0.0326 (17)	0.0268 (16)	0.0293 (17)	−0.0031 (14)	−0.0014 (14)	−0.0017 (13)
C19	0.0263 (15)	0.0217 (14)	0.0201 (14)	0.0024 (12)	−0.0011 (12)	−0.0033 (11)
C20	0.0233 (15)	0.0210 (14)	0.0221 (15)	−0.0002 (11)	0.0021 (12)	0.0014 (11)
C21	0.0276 (16)	0.0235 (15)	0.0282 (16)	0.0030 (12)	0.0007 (13)	−0.0014 (13)
C22	0.0349 (17)	0.0251 (16)	0.0372 (18)	0.0009 (13)	−0.0013 (15)	0.0000 (15)
C23	0.049 (2)	0.0245 (16)	0.0329 (18)	0.0110 (15)	0.0021 (17)	−0.0016 (15)
C24	0.045 (2)	0.039 (2)	0.043 (2)	0.0157 (17)	0.0108 (17)	0.0005 (17)
C25	0.0315 (19)	0.044 (2)	0.062 (3)	0.0031 (16)	0.0074 (18)	−0.0036 (19)
C26	0.0362 (19)	0.0271 (16)	0.0406 (19)	0.0022 (14)	0.0025 (16)	−0.0006 (15)

### Geometric parameters (Å, °)

**Table:**

S1—O1	1.425 (2)	C10—H10A	0.9900
S1—O2	1.434 (3)	C10—H10B	0.9900
S1—N1	1.610 (3)	C11—C12	1.525 (6)
S1—C1	1.777 (3)	C11—H11A	0.9900
S2—O5	1.428 (3)	C11—H11B	0.9900
S2—O4	1.434 (3)	C12—C13	1.534 (5)
S2—N3	1.604 (3)	C12—H12A	0.9900
S2—C14	1.777 (3)	C12—H12B	0.9900
O3—C7	1.252 (4)	C13—H13A	0.9900
O6—C20	1.242 (4)	C13—H13B	0.9900
N1—H11N	0.92 (4)	C14—C15	1.390 (4)
N1—H12N	0.86 (4)	C14—C19	1.397 (4)
N2—C7	1.320 (4)	C15—C16	1.390 (5)
N2—C8	1.467 (4)	C15—H15	0.9500
N2—H2N	0.91 (4)	C16—C17	1.385 (5)
N3—H31N	0.85 (4)	C16—H16	0.9500
N3—H32N	0.88 (4)	C17—C18	1.387 (5)
N4—C20	1.325 (4)	C17—H17	0.9500
N4—C21	1.470 (4)	C18—C19	1.393 (4)
N4—H4N	0.85 (4)	C18—H18	0.9500
C1—C2	1.390 (4)	C19—C20	1.501 (4)
C1—C6	1.405 (4)	C21—C26	1.518 (5)
C2—C3	1.382 (5)	C21—C22	1.525 (4)
C2—H2	0.9500	C21—H21	1.0000
C3—C4	1.371 (6)	C22—C23	1.525 (4)
C3—H3	0.9500	C22—H22A	0.9900
C4—C5	1.381 (5)	C22—H22B	0.9900
C4—H4	0.9500	C23—C24	1.513 (5)
C5—C6	1.393 (4)	C23—H23A	0.9900
C5—H5	0.9500	C23—H23B	0.9900
C6—C7	1.500 (4)	C24—C25	1.524 (5)
C8—C13	1.510 (5)	C24—H24A	0.9900
C8—C9	1.524 (4)	C24—H24B	0.9900
C8—H8	1.0000	C25—C26	1.521 (5)
C9—C10	1.531 (5)	C25—H25A	0.9900
C9—H9A	0.9900	C25—H25B	0.9900
C9—H9B	0.9900	C26—H26A	0.9900
C10—C11	1.498 (7)	C26—H26B	0.9900
			
O1—S1—O2	120.00 (16)	C11—C12—C13	110.1 (3)
O1—S1—N1	107.43 (16)	C11—C12—H12A	109.6
O2—S1—N1	106.70 (17)	C13—C12—H12A	109.6
O1—S1—C1	106.58 (14)	C11—C12—H12B	109.6
O2—S1—C1	107.23 (15)	C13—C12—H12B	109.6
N1—S1—C1	108.50 (16)	H12A—C12—H12B	108.1
O5—S2—O4	119.74 (18)	C8—C13—C12	110.9 (3)
O5—S2—N3	107.38 (17)	C8—C13—H13A	109.5
O4—S2—N3	106.62 (17)	C12—C13—H13A	109.5
O5—S2—C14	105.93 (15)	C8—C13—H13B	109.5
O4—S2—C14	107.90 (16)	C12—C13—H13B	109.5
N3—S2—C14	108.96 (17)	H13A—C13—H13B	108.0
S1—N1—H11N	114 (2)	C15—C14—C19	120.8 (3)
S1—N1—H12N	112 (3)	C15—C14—S2	118.6 (3)
H11N—N1—H12N	113 (4)	C19—C14—S2	120.5 (2)
C7—N2—C8	123.6 (3)	C16—C15—C14	120.3 (3)
C7—N2—H2N	117 (2)	C16—C15—H15	119.9
C8—N2—H2N	119 (2)	C14—C15—H15	119.9
S2—N3—H31N	112 (3)	C17—C16—C15	119.3 (3)
S2—N3—H32N	114 (3)	C17—C16—H16	120.4
H31N—N3—H32N	114 (4)	C15—C16—H16	120.4
C20—N4—C21	122.8 (3)	C16—C17—C18	120.4 (3)
C20—N4—H4N	118 (2)	C16—C17—H17	119.8
C21—N4—H4N	119 (2)	C18—C17—H17	119.8
C2—C1—C6	120.5 (3)	C17—C18—C19	121.0 (3)
C2—C1—S1	118.5 (2)	C17—C18—H18	119.5
C6—C1—S1	120.9 (2)	C19—C18—H18	119.5
C3—C2—C1	120.2 (3)	C18—C19—C14	118.2 (3)
C3—C2—H2	119.9	C18—C19—C20	118.8 (3)
C1—C2—H2	119.9	C14—C19—C20	122.8 (3)
C4—C3—C2	119.9 (3)	O6—C20—N4	123.8 (3)
C4—C3—H3	120.1	O6—C20—C19	119.9 (3)
C2—C3—H3	120.1	N4—C20—C19	116.3 (3)
C3—C4—C5	120.3 (3)	N4—C21—C26	111.3 (3)
C3—C4—H4	119.9	N4—C21—C22	108.6 (3)
C5—C4—H4	119.9	C26—C21—C22	110.9 (3)
C4—C5—C6	121.5 (3)	N4—C21—H21	108.6
C4—C5—H5	119.3	C26—C21—H21	108.6
C6—C5—H5	119.3	C22—C21—H21	108.6
C5—C6—C1	117.6 (3)	C23—C22—C21	111.7 (3)
C5—C6—C7	118.6 (3)	C23—C22—H22A	109.3
C1—C6—C7	123.6 (3)	C21—C22—H22A	109.3
O3—C7—N2	124.1 (3)	C23—C22—H22B	109.3
O3—C7—C6	120.5 (3)	C21—C22—H22B	109.3
N2—C7—C6	115.3 (3)	H22A—C22—H22B	107.9
N2—C8—C13	111.3 (3)	C24—C23—C22	111.5 (3)
N2—C8—C9	108.7 (3)	C24—C23—H23A	109.3
C13—C8—C9	111.3 (3)	C22—C23—H23A	109.3
N2—C8—H8	108.5	C24—C23—H23B	109.3
C13—C8—H8	108.5	C22—C23—H23B	109.3
C9—C8—H8	108.5	H23A—C23—H23B	108.0
C8—C9—C10	111.3 (3)	C23—C24—C25	110.7 (3)
C8—C9—H9A	109.4	C23—C24—H24A	109.5
C10—C9—H9A	109.4	C25—C24—H24A	109.5
C8—C9—H9B	109.4	C23—C24—H24B	109.5
C10—C9—H9B	109.4	C25—C24—H24B	109.5
H9A—C9—H9B	108.0	H24A—C24—H24B	108.1
C11—C10—C9	112.3 (3)	C26—C25—C24	110.5 (3)
C11—C10—H10A	109.1	C26—C25—H25A	109.6
C9—C10—H10A	109.1	C24—C25—H25A	109.6
C11—C10—H10B	109.1	C26—C25—H25B	109.6
C9—C10—H10B	109.1	C24—C25—H25B	109.6
H10A—C10—H10B	107.9	H25A—C25—H25B	108.1
C10—C11—C12	111.5 (3)	C21—C26—C25	110.8 (3)
C10—C11—H11A	109.3	C21—C26—H26A	109.5
C12—C11—H11A	109.3	C25—C26—H26A	109.5
C10—C11—H11B	109.3	C21—C26—H26B	109.5
C12—C11—H11B	109.3	C25—C26—H26B	109.5
H11A—C11—H11B	108.0	H26A—C26—H26B	108.1
			
O1—S1—C1—C2	134.7 (3)	O5—S2—C14—C15	133.5 (3)
O2—S1—C1—C2	5.0 (3)	O4—S2—C14—C15	4.1 (3)
N1—S1—C1—C2	−109.9 (3)	N3—S2—C14—C15	−111.3 (3)
O1—S1—C1—C6	−43.4 (3)	O5—S2—C14—C19	−42.3 (3)
O2—S1—C1—C6	−173.1 (3)	O4—S2—C14—C19	−171.7 (3)
N1—S1—C1—C6	72.0 (3)	N3—S2—C14—C19	72.9 (3)
C6—C1—C2—C3	−2.5 (6)	C19—C14—C15—C16	−0.7 (5)
S1—C1—C2—C3	179.4 (3)	S2—C14—C15—C16	−176.5 (3)
C1—C2—C3—C4	1.5 (6)	C14—C15—C16—C17	0.8 (5)
C2—C3—C4—C5	0.6 (6)	C15—C16—C17—C18	0.3 (5)
C3—C4—C5—C6	−1.7 (6)	C16—C17—C18—C19	−1.4 (5)
C4—C5—C6—C1	0.7 (5)	C17—C18—C19—C14	1.5 (5)
C4—C5—C6—C7	−174.5 (3)	C17—C18—C19—C20	−173.9 (3)
C2—C1—C6—C5	1.4 (5)	C15—C14—C19—C18	−0.5 (4)
S1—C1—C6—C5	179.4 (2)	S2—C14—C19—C18	175.3 (2)
C2—C1—C6—C7	176.4 (3)	C15—C14—C19—C20	174.8 (3)
S1—C1—C6—C7	−5.6 (5)	S2—C14—C19—C20	−9.5 (4)
C8—N2—C7—O3	0.7 (5)	C21—N4—C20—O6	3.6 (5)
C8—N2—C7—C6	177.3 (3)	C21—N4—C20—C19	−179.2 (3)
C5—C6—C7—O3	114.7 (3)	C18—C19—C20—O6	120.3 (3)
C1—C6—C7—O3	−60.2 (4)	C14—C19—C20—O6	−55.0 (4)
C5—C6—C7—N2	−62.1 (4)	C18—C19—C20—N4	−57.0 (4)
C1—C6—C7—N2	123.0 (3)	C14—C19—C20—N4	127.7 (3)
C7—N2—C8—C13	78.8 (4)	C20—N4—C21—C26	68.3 (4)
C7—N2—C8—C9	−158.2 (3)	C20—N4—C21—C22	−169.3 (3)
N2—C8—C9—C10	−177.0 (3)	N4—C21—C22—C23	−176.7 (3)
C13—C8—C9—C10	−54.0 (4)	C26—C21—C22—C23	−54.1 (4)
C8—C9—C10—C11	53.1 (5)	C21—C22—C23—C24	54.0 (4)
C9—C10—C11—C12	−54.6 (5)	C22—C23—C24—C25	−55.5 (4)
C10—C11—C12—C13	56.3 (5)	C23—C24—C25—C26	57.6 (4)
N2—C8—C13—C12	178.2 (3)	N4—C21—C26—C25	177.4 (3)
C9—C8—C13—C12	56.7 (4)	C22—C21—C26—C25	56.3 (4)
C11—C12—C13—C8	−57.4 (4)	C24—C25—C26—C21	−58.2 (4)

### Hydrogen-bond geometry (Å, °)

**Table:**

*D*—H···*A*	*D*—H	H···*A*	*D*···*A*	*D*—H···*A*
N1—H11N···O4^i^	0.92 (4)	2.05 (4)	2.935 (4)	161 (4)
N2—H2N···O6^ii^	0.91 (4)	1.96 (4)	2.874 (4)	175 (4)
N3—H32N···O2^iii^	0.88 (4)	2.23 (4)	2.943 (4)	138 (4)
C3—H3···O1^iv^	0.95	2.54	3.254 (4)	132.
N1—H12N···O3	0.86 (4)	2.18 (4)	2.938 (4)	146 (4)
N3—H31N···O6	0.85 (4)	2.09 (4)	2.831 (4)	145 (4)
N4—H4N···O3	0.85 (4)	2.11 (4)	2.952 (4)	171 (3)

Symmetry codes: (i) *x*, *y*+1, *z*; (ii) −*x*+1/2, *y*, *z*+1/2; (iii) −*x*+1/2, *y*−1, *z*−1/2; (iv) *x*−1/2, −*y*+1, *z*.
